# Advances in pharmacological strategies for the prevention of cataract development

**DOI:** 10.4103/0301-4738.49390

**Published:** 2009

**Authors:** S K Gupta, V Kalai Selvan, S S Agrawal, Rohit Saxena

**Affiliations:** Delhi Institute of Pharmaceutical Sciences and Research, Sector 3, Pushp Vihar, New Delhi-110 017, India; 1 RP Center for Ophthalmic Research, All India Institute of Medical Sciences, Ansari Nagar, New Delhi - 110 016, India

**Keywords:** Aldose reductase, antioxidants, glutathione, vitamins

## Abstract

Cataractous-opacification of the lens is one of the leading causes of blindness in India. The situation can be managed by surgical removal of the cataractous lens. Various pharmacological strategies have been proposed for the prevention and treatment of cataract. Information on possible benefits of putative anticataract agents comes from a variety of approaches, ranging from laboratory experiments, both *in vitro* and *in vivo*, to epidemiological studies in patients. This review deals with the various mechanisms, and possible pharmacological interventions for the prevention of cataract. The article also reviews research on potential anticataractous agents, including aldose reductase inhibitors, glutathione boosters, antiglycating agents, vitamins and various drugs from indigenous sources.

Cataract remains the leading cause of visual disability and blindness worldwide.[1] It is estimated that 180 million people are visually disabled globally. Of these, 37 million people are blind and this number increases by one to two million every year with 28,000 new cases reported daily.[2] Cataract contributes to 50% of blindness worldwide.[3] The proportion of blindness in children due to cataract varies considerably between regions from 10–30% with a global average estimated at 14%, leaving 190,000 children blind from cataract.[4] At present, the only remedy is surgical removal of the cataractous lens and substituting it with a lens made of synthetic polymers. However, the incidence is so large that the available surgical facilities are unable to cope up with the problem. In addition to these, postoperative complications can occur such as posterior capsular opacification, endophthalmitis and uncorrected residual refractive error.[5] Therefore, there is a search for pharmacological intervention that will maintain the transparency of the lens. During the last two decades, extensive research inputs have been made to delineate the etiology of cataract. Efforts have been directed to delay the onset and slow down the progression of cataract by various agents. Unfortunately, despite serious efforts, no single agent has proven clinically useful for this purpose. This review highlights the various pharmacological strategies for the prevention of cataract development and risk factors implicated in cataractogenesis.

## Factors Implicated in Cataractogenesis

Several risk factors have been identified in the pathogenesis of senile cataract. Apart from aging, smoking, diabetes, gender, steroids and nitric oxide are responsible for the development of cataract. These risk factors have been associated with different morphological types of cataract.

### Smoking:

Smoking is thought to increase the risk of cataract, at least in part, by increasing the oxidative stress in the lens caused by the generated free radicals. In the presence of tobacco smoke these free radicals may directly damage lens proteins and the fiber cell membrane in the lens.[6,7] Tobacco leaves contain a significant amount of cadmium (Cd), which is absorbed into the body when a person smokes or chews tobacco and this Cd replaces the bivalent metals like zinc (Zn), copper (Cu) and manganese from superoxide dismutase (SOD), a powerful antioxidant.[8]

### Diabetes:

There are several ways that diabetes can affect the eyes but the most common cause of loss of vision is cataract. Cataractogenesis is one of the earliest secondary complications of diabetes mellitus, a severe metabolic disorder characterized by hyperglycemia.[2] Some mechanisms have been proposed for cataract formation in diabetes mellitus such as excessive tissue sorbitol concentrations, abnormal glycosylation of lens proteins and increased free radical production.[9]

### Female gender:

A number of epidemiological studies using cross-sectional data have shown an increased prevalence of cataract in women compared with men.[10] The cause of the gender differences in cataract occurrence is not clear but could be related to the hormonal differences between women and men. Postmenopausal estrogen deficiency may be a factor. Recent epidemiologic data provided some evidence that estrogen and hormone replacement therapy may play a protective role in reducing the incidence of age-related cataract.[11]

### Steroids:

The association between steroid use and development of cataract is well established. There seems to be a consensus that higher the dose of steroid and longer the duration of use, the higher will be the risk for posterior subcapsular cataracts.[12] Steroids cause an inhibition of the cation pump in the lens capsule the resulting electrolyte/water imbalance is responsible for cataract formation.[13]

### Nitric oxide:

O_2_^−^ in itself is not highly toxic but it may react with other molecules yielding more reactive compounds. For example, the reaction with nitric oxide (NO) generates peroxynitrite (ONOO^−^), which causes extensive cell damage and can also have an important role in diabetic cataract formation.[14,15]

**Figure 1:**
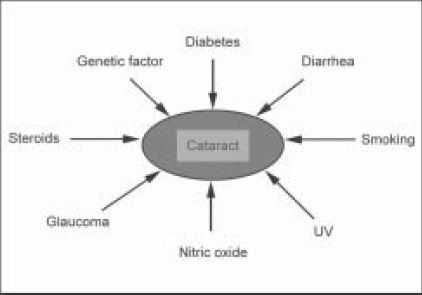
Major risk factors implicated in cataractogenesis

Apart from the above mentioned risk factors, genetic factors, socioeconomic status, illiteracy, malnutrition, diarrhea, myopia, renal failure, hypertension, sunlight, ultraviolet (UV) exposure, obesity, chemical burn, glaucoma and alcohol[16,17] have also been implicated in cataractogenesis [Fig. 1].

## Mechanisms Associated with Cataract

**Figure 2:**
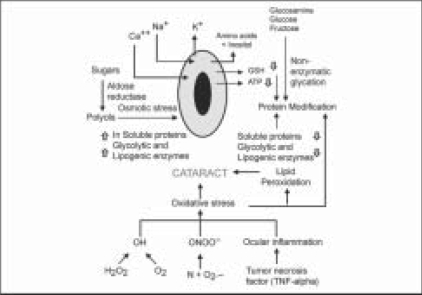
Mechanisms associated with cataractogenesis

Loss of transparency during human cataract formation results from a variety of complex metabolic and physiological mechanisms, which act in combination to change the refractive index.[18] Studies on lens proteins indicate that post-translational modifications occur in the lens proteins during cataractogenesis as a result of chemical actions that include oxidation, glycation, Schiff base formation, proteolysis, transmidation, carbamylation, phosphorylation and elevated calcium levels[17] [Fig. 2]. The post-translational modifications alter attractive forces between lens proteins to favor aggregation, disruption of normal lens cell structure and opacification.[2]

Among the multiple mechanisms proposed for cataractogenesis this article explains the role of the following pathways in cataract development.

- Non-enzymatic glycation
- Oxidative stress
- Polyol pathway

### Non-enzymatic glycation:

**Figure 3:**
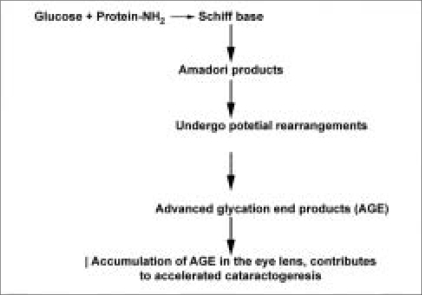
Formation of advanced glycation end products

Under hyperglycemic conditions, part of the excess glucose reacts non-enzymatically with proteins or other tissue or blood constituents, thus increasing the physiological rate of non-enzymatic glycation.[19] Chronic, irreversible abnormalities unaffected by normalization of blood glucose levels primarily involve long-lived molecules, extracellular matrix, eye lens crystallins, and chromosomal DNA. Due to their characteristic chemical properties, advanced products of non-enzymatic glycation play a critical role in the evolution of sugar cataract. The formation of advanced glycation end products (AGEs) begins with the attachment of a glucose carbonyl group to a free amino group of proteins or amino acids to form a labile Schiff base adduct as the first step of the complex Maillard process. Levels of the unstable Schiff base increase rapidly, and equilibrium is reached after several hours. Once formed, Schiff base adducts undergo a slow chemical rearrangement over a period of weeks to form a more stable, but still chemically reversible, Amadori product[20] [Fig. 3].

Specific chemical characterization of AGE proteins has been difficult, as Amadori products can theoretically undergo a large number of potential rearrangements. Immunological and chemical evidence indicates that progressive accumulation of AGEs in the diabetic eye lens contributes to accelerate cataractogenesis in hyperglycemic experimental animals and diabetic humans.[21,22]

### Oxidative stress:

The osmotic and exogenous or endogenous oxidative stresses play an important role in the pathogenesis of cataract.[23] Oxidative stress may result from an imbalance between the production of reactive oxygen species (ROS) and the cellular antioxidant defense mechanisms. In the cells of the eyes, ROS may initiate a surge of toxic biochemical reactions such as peroxidation of membrane lipids and extensive damage to proteins causing intracellular protein aggregation and precipitation and eventually leading to lens opacification.[24,25] On exposure of the eye to oxidative stress, the redox set point of the single layer of the lens epithelial cells quickly changes, going from a strongly reducing to an oxidizing environment. Almost concurrent with this change is extensive damage to the DNA and membrane pump systems, followed by loss of epithelial cell viability and death by necrotic and apoptotic mechanisms leading to cataract.[26,27]

### Polyol pathway:

**Figure 4:**
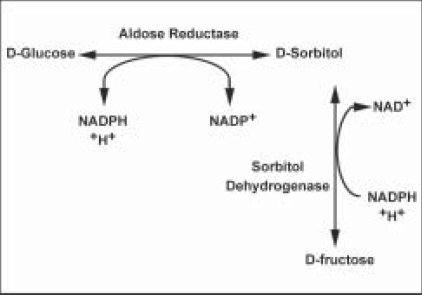
Polyol pathway

The mechanism involved in the progression of diabetic cataracts is different from senile cataracts. The accumulation of polyols within the lens is the primary contributing factor. Certain tissues of the body, including the eye lens, do not require insulin for glucose and other simple sugars to enter. In diabetes, sugar is in high concentration in the aqueous humor and can diffuse passively into the lens. The enzyme aldose reductase within the lens converts glucose to sorbitol or galactose to galactitol [Fig. 4]. These polyols cannot diffuse passively out of the lens and accumulate or convert to fructose. The accumulation of polyols results in an osmotic gradient, which encourages diffusion of fluid from the aqueous humor. The water drags sodium with it and the swelling and electrolyte imbalances result in cataract formation.

## Pharmacological Strategies for Prevention of Cataract

Drugs have been developed which are aimed to interact at the level of altered lens metabolism and lens pathophysiology. The anticataract agents claimed to be effective *in vitro, in vivo* and in epidemiological studies may be broadly classified in the following categories:

- Aldose reductase inhibitors
- Non-steroidal anti-inflammatory drugs
- Agents acting on glutathione
- Vitamins, minerals, antioxidants and herbal drugs
- Miscellaneous agents

### Aldose reductase inhibitors (ARI):

ARI are aimed to block the metabolic pathways of glucose responsible for diabetic vascular dysfunction. Their role in the prevention of diabetic cataract in animals is now well established.[28,29] Numerous natural and synthetic compounds have been found to inhibit aldose reductase. These so-called ARI bind to aldose reductase, inhibiting polyol production. The rationale of using sorbitol-lowering agents has eroded over the years because the enzyme aldose reductase is remarkably sluggish with glucose. Furthermore, adult human lenses incubated in high glucose media do not accumulate sorbitol. There are a number of ARI known to possess anticataract potential and delay the galactose-induced cataract in different experimental models.[30] Some of these include alrestatin, sorbinil, sulindac, naproxen, aspirin, tolrestat, statil and bioflavonoids.

Flavonoids are among the most potent naturally occurring ARI. Several evaluations of *in vitro* animal lenses incubated in high-sugar mediums have found flavonoids to inhibit aldose reductase.[31,32] In our previous studies the flavonoids quercetin and myricetin have shown significant delay in the onset and progression of galactose cataract in rats.[33] The flavonoids quercetrin and quercetrin-2-acetate, quercetin, rutin, hesperidin, hesperidin chalcone and naringin exhibited AR-inhibiting activity to different extents.[34] A recent study was carried out to evaluate the potential of AR-inhibiting bioflavonoids extracted from the fruits of *G. applanatum*.[35]

A recent study by Varma *et al.*, demonstrated that administration of pyruvate prevented cataract development by inhibiting the AR in diabetic rats.[37] The AR-inhibiting activity in *Emblica officinalis* was investigated and found to be better than quercetin.[38] Similarly, vitamin C also has potential as an ARI with both animal and clinical studies showing that it minimizes the sorbitol levels.[38,39] The aqueous extract of *Gymnema sylvestre* showed potential AR inhibition in sugar-induced cataract and also protected the lens from osmotic damage.[40]

Table 1 demonstrates some of the most commercially available flavonoids and herbal drugs and their comparative inhibitions.

**Table 1:** Aldose reductase inhibiting activity of some flavonoids

Flavonoid	Percent inhibition
Quercetrin-2-acetate[32]	100
Quercetrin[32]	100
Quercetin[32]	100
Rutin[32]	95
Hesperidin[32]	88
Hesperidin chalcone[32]	82
Naringin[32]	80
Emblica officinalis[37]	82.4

Among the ARI only sorbinil reached the advanced clinical trial stages in cataract prevention program. However, due to manifestation of skin rashes the trial had to be discontinued. In spite of extensive research input, clinical trials of the sorbitol-lowering agents have not produced convincing proof of their efficacy.

### Non-steroidal anti-inflammatory drugs:

Non-steroidal anti-inflammatory drugs (NSAIDs) have emerged as another group of drugs with anticataract potential. The first indication regarding the probable use of NSAIDs as prophylactic anticataract agents came from studies on aspirin use in patients with rheumatoid arthritis and diabetes.[41] Subsequently, a number of NSAIDs with diverse chemical structures were reported to delay the phenomenon in experimental animals. The NSAIDs extensively studied are aspirin, paracetamol, ibuprofen, naproxen, sulindac and bendazec.[42–44] The anticataract activity of these drugs is explained by virtue of their effect on different biochemical pathways. The mechanisms associated with the protective effect of NSAIDs include acetylation, inhibition of glycosylation and carbamylation of lens proteins.[42]

We have earlier shown that naproxen delays the onset and progression of galactose-induced cataract in rats.[45] *In vivo* effectiveness of naproxen has also been tested in rat pups developing cataracts under oxidative influence of sodium selenite.[46] To elucidate the mechanism of action of naproxen as an antioxidant, its effect on light-induced lipid peroxidation in isolated rat lenses was studied and depletion of lens glutathione and rise in malondialdehyde levels was observed.[47] It was also shown that sulindac inhibits lens polyol to a great degree by its possible inhibitory action on lens AR.[48] Comparative studies on the anticataract activity of various NSAIDs revealed that though inhibition of lens AR by NSAIDs could be a significant factor it does not seem to be the sole cause.[45] The hypothesis of acetylation of lens protein by aspirin does not justify the mechanism of action of other NSAIDs like ibuprofen, which do not have acetyl group. The results obtained so far indicate that there are multiple sites where NSAIDs probably act and prevent cataract progression. However, there is a need to explore their mechanisms of action in more detail under different culture conditions and in different experimental models.

Anticataract activity of aspirin, sulindac, and naproxen eye drops was also studied and they were found to delay both onset and progression of cataract in different models of cataractogenesis, moreover, there were no adverse side-effects even after long-term application.[49] Subsequent studies further confirmed that aspirin is a potential anticataract agent.[50]

Bendazac, a compound resembling indomethacin in its structure, emerged as a potential radical scavenger and anticataract agent. Bendazac protects lens and serum proteins' denaturation *in vitro* and *in vivo*.[51,52] 5-hydroxybendazac, a derivative, was found to be more effective than the parent compound in protecting lens proteins against cyanate, glucose-6-phosphate and galactose.[53] Another derivative, bendazac-lysine was found to have better absorption in animal and human studies and it is reported to delay cataractogenesis.[54] Bendazac-lysine has undergone clinical trials but these studies have been small and of short duration.[55] Bendazac-lysine is already available as an anticataract drug in Italy and in several other European countries under the trade name Carbopol 980NF manufactured by Goodrich Limited.

### Agents which act on glutathione:

**Figure 5:**
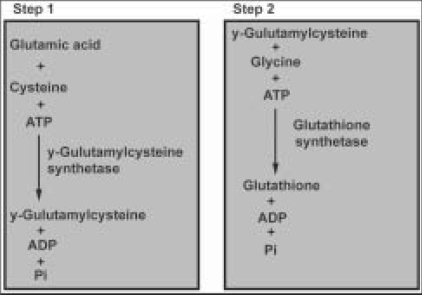
Synthesis of glutathione within the lens

The most important function of glutathione (GSH) is to deactivate and render excess free radicals and keep them harmless. GSH is composed of the amino acids cysteine, glutamic acid, and glycine, and its synthesis within the lens takes place in two ATP-dependent steps [Fig. 5]. There are several ways in which GSH or its depletion can affect the opacity of the lens. A review by researchers on GSH[56] the mechanisms of cataract prevention is: (1) maintaining sulfhydryl (SH) groups on proteins in their reduced form preventing disulfide cross-linkage; (2) protecting SH groups on proteins important for active transport and membrane permeability; and (3) preventing oxidative damage from H_2_ O_2_.

The concentration of GSH decreases with age in the lens and more markedly in cataract.[34] GSH has been reported to control calcium influx and protect lens protein against damaging effects of osmotic and oxidative stress.[5,57] Large amount of research has been done on antioxidants and vitamins; and the role of GSH in the prevention of cataract has been reported. A recent study indicates that vitamin E protects the anti-oxidative defense mechanism directly or indirectly through increased levels of GSH.[58] The anticataract effect of melatonin (a scavenger of free radical), was demonstrated and the study concluded that the effect is due to its stimulatory effect on GSH production.[59]

Clinical trials on Phaken, a preparation containing three constituents of amino acids GSH plus arginine, inostitol, pyridoxine and ascorbic acid have shown to improve visual acuity, but due to a high dropout rate no clear-cut conclusions could be drawn.[60]

## Vitamins, Minerals, Antioxidants and Herbal Drugs

### Vitamins:

The potential role of vitamins in preventing cataract is well documented, especially vitamin C or ascorbic acid which plays an important part in lens biology, both as an antioxidant and as a UV filter.[61] Dietary deficiency of vitamin C led to reduction in lens concentrations of ascorbate.[62] A research study on guinea pigs shows that ascorbate inhibits galactose cataract.[63] Similarly, another study reveals that intake of ascorbate increases the level of vitamin C in rat lens.[64] Vitamin E also has an important part to play in lenticular antioxidant status. A number of studies have evaluated the anticataract potential of vitamin E and found it to be effective against galactose, steroid and UV radiation-induced cataract.[58,65–67] Riboflavin is a precursor to flavin adenine dincleotide (FAD), which is a coenzyme for the biosynthesis of glutathione reductase. *In vitro* evaluations of surgically removed cataracts have confirmed inactivity of glutathione reductase enzyme activity in a significant number of cataracts examined.[68] Furthermore, the activity was restored by the addition of FAD. A study of B vitamin nutritional status of cataract patients (n = 37) compared to age-matched controls without cataract (n = 16) found that 80% of those with cataracts and only 12.5% of control subjects had a riboflavin deficiency.[69]

### Minerals:

The excessive free radical attack implicated in the development of cataract can be prevented by dietary intake of micronutrients such as zinc, copper and manganese. Copper and zinc are required for the catalytic activity of metal protein and SOD.[70] Plasma levels of zinc and copper were found to be significantly low in cataract patients.[69] Selenium is an integral part of the enzyme, glutathione peroxidase. A decrease in glutathione peroxidase activity has been found in the lenses of selenium-deficient rats.[71]

### Antioxidants:

It is widely accepted that oxidative stress is a significant factor in the progression of cataractogenesis.[72–74] Oxidative stress is associated with increased reactive oxygen species and is known to accelerate cataract formation since superoxide is converted to a toxic substance, namely hydrogen peroxide. This reaction is prevented by antioxidant enzymes, namely catalase, superoxide dismutase and glutathione peroxidase. Antioxidants are a key prophylactic agent in preventing oxidation-related cataractogenesis. A large number of epidemiological and interventional studies have been investigated for the role of dietary antioxidant supplement in the incidence of cataract.

Carotenoids are natural lipid-soluble antioxidants. It is reported that persons with a high intake of carotene reduce the incidence of risk of cataract[75] and the relationship between nuclear cataract and intakes of α-carotene, β-carotene, lutein, lycopene and cryptoxanthin stratifying by gender and by regular multivitamin use.[76] Amongst all carotenoids lycopene has a high antioxidative activity and exerts a protective effect in various diseases.[77] In previous studies, we found that lycopene protects against oxidative stress-induced experimental cataract[74] and prevented sugar-induced diabetic cataract.[57]

Curcumin, the active principle of turmeric, has been shown to have antioxidant activity *in vitro* and *in vivo*.[78] The effect of curcumin on cataract has also been established. Curcumin delayed the onset and maturation of galactose-induced[79] and streptozotocin-induced diabetic cataracts.[80] Curcumin also prevented oxidative stress-induced cataract.[81]

Numerous studies proved stobadine, a novel synthetic pyridoindole, to be an efficient antioxidant[82] and *in vitro* it was found to protect bovine serum albumin against glycol-oxidative damage.[83] Stobadine has been shown to delay the development of diabetic cataract.[84]

### Herbal drugs:

In recent years, a great emphasis has been laid on exploring the possibility of using our natural resources to delay the onset and progression of cataract. A great number of medicinal plants and their formulations are reported to possess antioxidant properties and offer protection against cataract.

Gupta *et al.*, have shown that the aqueous extract of *Ocimum sanctum* possesses potential anticataract activity against oxidative stress-induced experimental cataractogenesis. The protective effect was supported by restoration of the antioxidant defense system.[85] The aqueous extracts of well-known herbal antidiabetic drugs namely *Pterocarpus marsupium* and *Trigonella foenum-graceum* exerted a favorable anticataract effect.[86] A recent research study found that grape seed proanthocyanidin extract effectively suppressed cataract formation in rats.[87] Flavonoids from *Emilia sonchifolia* modulate the lens opacification and oxidative stress in selenite-induced cataract.[88] *Dregea volubilis* is a traditionally used medicinal plant for the treatment of various eye ailments; now its potential anticataract effect has been proved scientifically and it has also been found that the effect is due to drevogenin D, a triterpenoid aglycone.[89] *Vaccinium myritillus* or *bilberry* also has a long history of use for various eye conditions.[34] In a clinical study report of 50 patients with senile cataracts, a combination of bilberry and vitamin E stopped the progression of cataracts up to 96%[90]

Certain herbal drugs, especially *Ginkgo biloba* extract have been found to possess potential therapeutic effect in radiation-induced cataract.[91] The anticataract activity of Green tea (*Camellia sinensis*) has been studied extensively and the explained antioxidative potential is the major mechanism in the prevention of cataractogenesis. Gupta *et al*., have shown that green tea protects against selenite-induced cataract and acts primarily by preserving the antioxidant defense system.[92] It was also shown that the oxidative potential of green tea retards the progression of cataractogenesis.[93] Recently, both green tea and black tea have been demonstrated to retard the development of diabetic cataract also by hypoglycemic effect.[94] A recent study found that *E. officinalis*, commonly known as *amla*, used against diabetes, is also effective in delaying the progression of diabetic cataract.[95]

The herbal formulation Diabecon (used for diabetics, contains 25 herbal drugs) inhibited the sugar-induced lens opacity in organ culture and also demonstrated that the effect is mainly due to *Gymnema sylvestre*, which is one of its constituents.[40] A study by our laboratory on polyherbal preparation, Chyavanprash (containing about 35 natural herbs including amla), found it to be protective against steroid-induced opacities in lens of chick embryo.[96]

Table 2 summarizes potential vitamins, antioxidants and herbal drugs for the prevention and treatment of cataract.

**Table 2:** Vitamins, antioxidants and herbal drugs for the prevention and treatment of cataract

Supplement	Mechanism of action
Vitamin C[62,64]	Preserves glutathione levels; protects the Na+/K+pump.
Riboflavin[69]	Precursor to FAD, a coenzyme for glutathione reductase which recycles glutathione
Vitamin E[90]	Antioxidant; increases glutathione; supplementation associated with prevention
Glutathione[56]	Deficiency noted in cataractous lenses; important component of the innate antioxidant system in the lens
Carotenes[76]	Antioxidant; higher levels associated with decreased risk for cataract
Lycopene[77]	Major carotenoid; possesses potential antioxidative property; reduces the risk associated with osmotic stress
Curcumin[78,79]	Antioxidant; reduces apoptosis in sugar cataract; inhibits the enzyme AR in polyol pathway
Stobadine[84]	A novel synthetic pyridoindole, an antioxidant, effective against diabetic cataract
Ocimum sanctum[85]	Restores the antioxidant defense system; inhibits lens protein degradation
Emilia sonchifolia[88]	Acts as an antioxidant and inhibits the lipid peroxidation reaction
Emblica officinalis[95]	Potent inhibitors of AR; reduces the osmotic stress
Dregea volubilis[89]	Preserves the antioxidant mechanisms and lower the level of lipid peroxidation
Vaccinium myritillus[34]	Potent antioxidant
Ginkgo biloba[91]	Antioxidant that protects the lens from various oxidative damage
Camellia sinensis (green tea)[92]	Inhibits oxidative stress by balancing the antioxidant defense mechanisms
Pterocarpus marsupium[86]	Prevents diabetic cataract by reduces the risk associated with osmotic stress
Trigonella foenum-graceum[86]	Prevents diabetic cataract by reduceing the risk associated with osmotic stress
Grape seed[87]	Increases glutathione level; reduces the lipid peroxidation

## Miscellaneous Agents

Various substances with diverse chemical structures and properties have been found to have protective effect against cataract in different experimental models. A study was conducted with pyruvate, a compound of metabolic origin and possessing an alpha-keto-carboxyl group. It was found effective in delaying cataract formation in diabetic[97] as well as in selenite[98] models of experimental cataracts. A study also performed with alpha-ketoglutarate and was found to have a very substantial anticataratogenic property in selenite induced cataract.[98]

ACE inhibitors have found to afford protection from free radical damage in many experimental conditions.[99] Recently, the anticataract activity of lisinopril and enalapril was evaluated in glucose-induced cataract *in vitro* and found to offer significant protection. The study concluded that the effect might be because of the antioxidant and free radical scavenging activity, as evidenced by a decrease in malondialdehyde in the treated lens.[100]

N-acetylcarnosine (available as the ophthalmic drug Can-C) has been found to be effective in the prevention and treatment of age-related cataracts. It protects the crystalline lens from oxidative stress, and in a recent clinical trial it was shown to produce an effective, safe and long-term improvement in sight. When Can-C is administered topically, N-acetylcarnosine functions as a time-release prodrug form of L-carnosine, resistant to hydrolysis with carnosinase. N-acetylcarnosine has potential as an *in vivo* universal antioxidant because of its ability to protect against oxidative stress in the lipid phase of biological cellular membranes and in the aqueous environment by a gradual intraocular turnover into L-carnosine. The clinical effects of a topical solution of Can-C on lens opacities were examined in patients with cataracts and in canines with age-related cataracts. These data showed that N-acetylcarnosine is effective in the management of age-related cataract reversal and prevention both in human and in canine eyes.[101]

Protective effect of alpha lipoic acid,[102] pantethine,[103] DL-penicillamine[104] and deferoxamine[105] has been reported long back and unfortunately none of these drugs have been evaluated clinically. Various anticataract drugs like Itone (combination of 19 herbal drugs including triphala and tulsi) and a few herbal drugs are available in India without any proof of their efficacy, hence detailed scientific studies are required to ascertain the efficacy of these herbal drugs.

Studies on anticataract drugs are advancing on a number of fronts and a few drugs have reached the stage of clinical trials. Various groups are also trying to investigate the anticataract effect of drugs from natural and synthetic origin. Our preliminary studies have shown encouraging results on the use of a special combination of a few antioxidants and herbal drugs. It seems likely that some of these compounds will be shown in future to be effective in delaying or slowing the development of cataract.

## Conclusion

Much research has been done on epidemiological, *in vitro* and *in vivo* studies of vitamins, minerals, herbal drugs and nutritional supplementation in the prevention and treatment of cataracts. Although there are several drugs that may have potential for the treatment of cataract, most studies are merely preliminary. However, larger and prospective clinical studies on the use of nutrients and herbal drugs for the treatment of cataract are needed. Similarly, the possibility of toxicity associated with long exposure to most of the drugs limits this intervention. There are positive reports on topical use of these drugs with no or minimum side-effects. A prevention or delay through such an application in humans will prove to be a significant achievement against the cataract blindness in the world.
